# Delayed hepatic artery pseudoaneurysm with arterioportal fistula after penetrating liver trauma successfully treated with coil embolization: a case report and focused literature review

**DOI:** 10.3389/fsurg.2026.1803047

**Published:** 2026-07-14

**Authors:** Alhareth M. Amro, Mohammad Harbi Alfrookh, Salahaldeen Deeb, Yara Juma, Rahaf Masri, Baraa M. AbuMandil, Waled Sabarna, Rajai Alhusseini

**Affiliations:** 1 Faculty of Medicine, Al-Quds University, Jerusalem, Palestine; 2 Department of Vascular Surgery, Al-Ahli Hospital, Hebron, Palestine

**Keywords:** coil embolization, endovascular embolization, gelfoam, hepatic artery pseudoaneurysm, hepatic injury, penetrating liver trauma, vascular complication

## Abstract

Hepatic artery pseudoaneurysm (HAP) with arterioportal fistula (APF) is a rare but potentially life-threatening vascular complication of hepatic trauma, most commonly described after blunt or iatrogenic injury and infrequently following penetrating trauma. Early identification and prompt endovascular management are essential to prevent severe outcomes. We report the case of a 25-year-old previously healthy male who sustained a stab wound to the right upper quadrant and epigastrium and underwent exploratory laparotomy for a grade III left hepatic lobe laceration. During postoperative recovery, he developed persistent blood-tinged drain output and epigastric pain. Contrast-enhanced CT and subsequent angiography confirmed a left hepatic artery pseudoaneurysm with an associated arterioportal fistula. Initial Gelfoam embolization failed, but definitive coil embolization via the femoral artery successfully excluded the lesion and resolved the fistulous communication. The patient experienced symptomatic improvement and was discharged in stable condition. This case underscores the importance of recognizing delayed vascular complications following penetrating liver trauma and highlights endovascular coil embolization as a safe and effective therapeutic approach. Structured postoperative follow-up remains crucial for optimal outcomes.

## Introduction

The liver is one of the most commonly affected organs in cases of trauma, whether blunt or penetrating. This tendency is due to its location, large size, and high vascularity. A stab wound is found in 40% of liver injuries requiring laparotomy. In severe trauma, liver injury is followed by mortality, accounting for 10%–15% of the total (1). Due to medical advances, attempts are increasingly directed toward treating such injuries without surgery, particularly in elderly patients, children, or cases where hemodynamics is stable. Therefore, a clinical assessment is an important step in determining the severity of the injury, the patient's clinical status, and the presence of other injuries. All of these factors contribute to making an appropriate decision based on the patient's condition (2).

Hepatic pseudoaneurysm occurs when an artery loses its integrity due to atherosclerosis or a trauma to the liver, whether blunt or penetrating. This results in blood entering between the two layers of the blood vessels (tunica media and tunica adventitia), forming a pulsating mass in the artery (3). Its occurrence is rare, but its prevalence is not known with certainty. However, some reports indicate that it is 0.001% (4). despite rarity, is a serious complication, as it is usually discovered after it ruptures and the patient goes into shock due to massive blood loss. It is possible that the patient may not show symptoms if it is discovered early during routine follow-up after trauma. It's anatomically divided into arteries where the incidence of aneurysm is 85.2%, compared to 14.8% in extrahepatic (3).

Although numerous studies have demonstrated the occurrence of pseudoaneurysms after blunt trauma, few cases have been published after penetrating one (5, 6).

This may be due to the fact that penetrating patients are taken to the operating room, where any source of bleeding is stopped and any arterial abnormalities are addressed immediately. In contrast, cases following blunt trauma can be missed due to conservative treatment that may miss small arteries.

In this case study, we present a case of pseudoaneurysm following penetrating trauma with a knife. By publishing this case, we add to the small number of previously published cases following penetrating trauma. This aims to draw the attention of physicians to the importance of such cases and to address them early to avoid potential complications. This case report was prepared in accordance with the CARE reporting guidelines.

## Case presentation

A 25-year-old previously healthy male sustained a stab wound to the right upper quadrant and epigastrium in June 2025. He was immediately transferred to another hospital, where he underwent an urgent exploratory laparotomy. Intraoperative findings revealed a grade III laceration of the left hepatic lobe, which was repaired surgically. Two intra-abdominal drains were placed, and he received two units of packed red blood cells (PRBCs). The early postoperative course in the SICU was notable for persistent blood-tinged drain output (500 mL/day from the left drain), feeding intolerance with vomiting, and continuous epigastric and right upper quadrant pain. He remained hemodynamically stable, with preserved bowel and urinary function.

A contrast-enhanced abdominal CT scan performed two days after admission demonstrated an irregular hyperdense lesion measuring 2 × 1.5 cm, with surrounding minimal fluid in segments II/III of the left hepatic lobe, in proximity to a distal left portal vein branch. The impression was arteriovenous fistula vs. pseudoaneurysm. Serial abdominal ultrasounds showed no interval change in lesion size (Figure 1).

**Figure 1:**
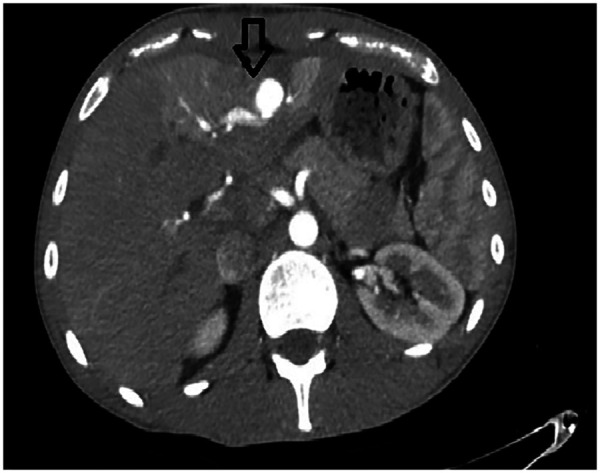
Contrast-enhanced abdominal CT scan showing a hyperdense lesion in segments II and III of the left hepatic lobes, adjacent to a distal left portal vein branch. The appearance is suggestive of a hepatic artery pseudoaneurysm with possible arterioportal fistula.

Although the initial CT raised suspicion for a vascular lesion, immediate transarterial angiography and definitive embolization were not performed at the referring hospital because interventional radiology resources were not immediately available. The patient was therefore managed initially with close clinical and radiologic observation. However, because epigastric pain and blood-tinged drain output persisted, an embolization attempt with Gelfoam was subsequently performed, but it failed to exclude the lesion.

The patient's vomiting gradually resolved, but he continued to experience postprandial epigastric pain. His right abdominal drain was removed, and he was transferred to the surgical ward on postoperative day five. He subsequently underwent percutaneous transhepatic embolization with Gelfoam, which failed. The patient left against medical advice and later presented to our hospital.

On presentation to our hospital, the patient was hemodynamically stable and afebrile. Physical examination showed a healing midline laparotomy wound without signs of infection. The abdomen was soft, with localized epigastric tenderness but no guarding or peritoneal signs. The remaining abdominal drain had minimal output. Laboratory testing showed mild anemia and mild hepatic enzyme abnormalities, while bilirubin, renal function, and coagulation parameters were preserved (Table 1).

**Table 1:** Laboratory investigations on admission.

Test	Result	Reference range	Interpretation
Albumin	3.4 g/dL	3.5–5.0	Slightly low
Alkaline phosphatase	155 U/L	40–129	Mildly elevated
Hemoglobin	9.1 g/dL	13–17	Anemia
ALT	166 U/L	<45	Significantly elevated
AST	51 U/L	<40	Mildly elevated
Bilirubin, total	0.7 mg/dL	0.2–1.2	Normal
BUN	10 mg/dL	7–20	Normal
Creatinine	0.57 mg/dL	0.6–1.3	Slightly low
PT	12.5 s	11–14	Normal
PTT	25 s	23–35	Normal

Laboratory values are presented as reported in the submitted case material. ALT, alanine aminotransferase; AST, aspartate aminotransferase; BUN, blood urea nitrogen; PT, prothrombin time; PTT, partial thromboplastin time.

A repeat contrast-enhanced CT abdomen confirmed a left hepatic artery pseudoaneurysm with arterioportal fistula, along with a few liver lacerations/hematomas and a small subcapsular peri-hepatic hematoma adjacent to the caudate segment. Angiography demonstrated a patent aorta and a pseudoaneurysm arising from branches of the left hepatic artery (segments II–III), with an arterioportal fistula involving the left portal vein and hepatic vein (Figures 2, 3). He underwent successful endovascular coil embolization via the right femoral artery, targeting the pseudoaneurysm and arterioportal fistula. Post-embolization angiography confirmed successful exclusion of the pseudoaneurysm and resolution of the fistulous communication, with no residual contrast extravasation (Figure 4).

**Figure 2:**
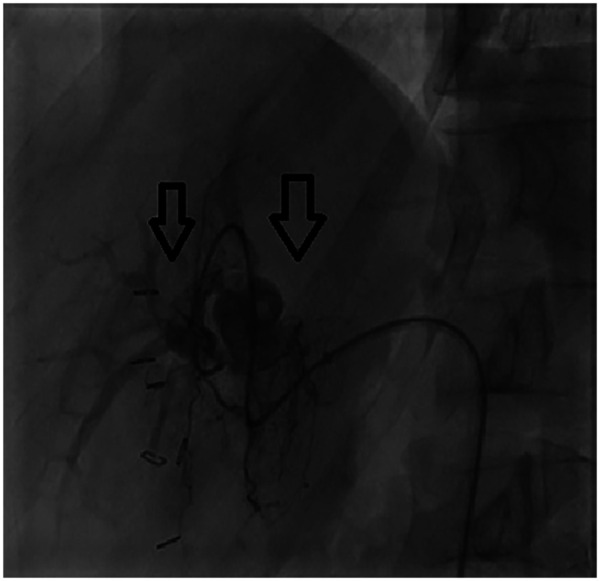
Digital subtraction angiography (DSA) confirming a pseudoaneurysm arising from the left hepatic artery with associated arterioportal fistula. Abnormal contrast opacification of the portal vein is observed during the arterial phase.

**Figure 3:**
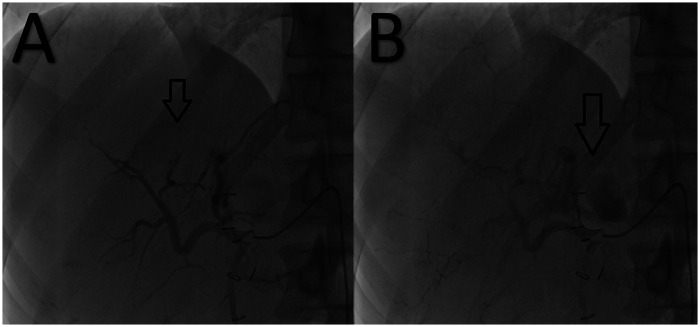
**(A)** selective angiography demonstrating arterial filling of the left hepatic artery branch proximal to the pseudoaneurysm. **(B)** Angiographic image showing the pseudoaneurysm and early portal venous filling, consistent with an arterioportal fistula.

**Figure 4:**
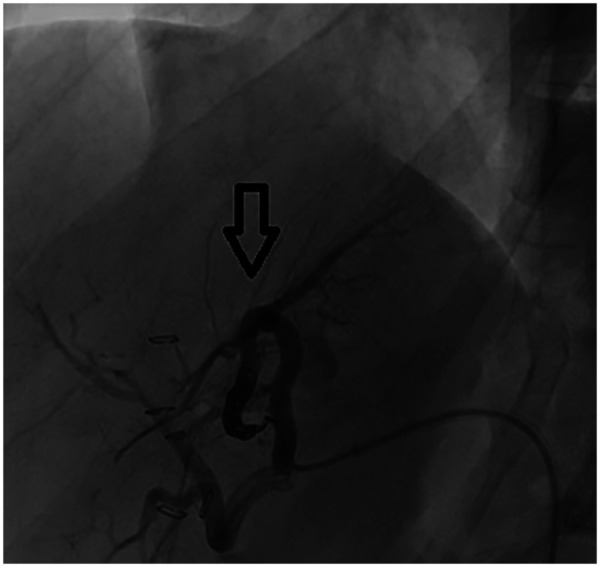
Post-embolization angiography demonstrating successful coil embolization of the pseudoaneurysm and exclusion of the arterioportal fistula, with no residual contrast extravasation.

The procedure was performed under general anesthesia. Right common femoral arterial access was obtained using a 6-Fr vascular sheath. Systemic heparinization was administered. Selective catheterization of the celiac trunk and common hepatic artery was performed followed by superselective catheterization of the left hepatic arterial branch. Angiography confirmed a pseudoaneurysm arising from segment II/III branches of the left hepatic artery with early venous filling consistent with arterioportal fistulous communication. Embolization was performed. achieving complete exclusion of the pseudoaneurysm and closure of the fistulous communication. Completion angiography demonstrated no residual pseudoaneurysm filling, no persistent fistulous shunting, and no contrast extravasation.

Following the intervention, the patient's abdominal pain improved, vomiting resolved, and drain output decreased. As a follow-up patient, we monitored post-abdominal trauma and liver injury, following surgical intervention and endovascular coiling. He remains stable, conscious, and oriented, with no abdominal tenderness and clear chest sounds. His vital signs and lab results including CBC, creatinine, electrolytes, and blood sugar, are within normal limits. The absence of drain output and adequate urine production suggests good recovery. The medical team continues to observe him closely to ensure no complications arise, maintaining conservative management and supportive care. He was discharged in improved condition with instructions for vascular surgery follow-up.

Follow-up CT performed, and the report showed no residual pseudoaneurysm filling, no persistent arterioportal shunting, and no new intra-abdominal collection. The patient remained clinically stable without recurrent abdominal pain, bleeding, or drain output.

## Discussion

This case presents a rare vascular complication in a previously healthy 25-year-old male who sustained a penetrating stab wound to the right upper quadrant and epigastrium. He underwent an urgent exploratory laparotomy at an outside facility, where a grade III laceration of the left hepatic lobe was surgically repaired. Despite initial postoperative hemodynamic stability, the patient developed persistent blood-tinged drain output and postprandial epigastric pain. A contrast-enhanced CT scan revealed a hyperdense lesion in segments II and III of the liver, suggestive of a hepatic artery pseudoaneurysm (HAP) with an arterioportal fistula (APF). Angiography confirmed these findings. After an unsuccessful attempt at embolization with Gelfoam, the patient was referred to our facility, where successful endovascular coil embolization was performed. The patient experienced symptom resolution and was discharged in a stable condition, emphasizing the importance of early recognition and appropriate intervention in such cases.

A hepatic artery pseudoaneurysm is a false aneurysm that results from a breach in the arterial wall, with blood dissecting into the surrounding tissue while remaining in communication with the arterial lumen. Unlike true aneurysms, which involve dilation of all three layers of the arterial wall (intima, media, and adventitia), pseudoaneurysms lack these structural components and are confined by adjacent tissues (7). Pseudoaneurysms can remain asymptomatic or may rupture, leading to life-threatening hemorrhage. In the liver, they often involve branches of the hepatic artery and may be complicated by arteriovenous or arterioportal fistulae, depending on the adjacent structures affected (8).

The etiology of hepatic artery pseudoaneurysms is diverse. Common causes include iatrogenic injury during hepatobiliary procedures (e.g., liver biopsy, cholecystectomy, and biliary drainage), blunt abdominal trauma, penetrating trauma, and infections such as hepatic abscesses (3). Inflammatory conditions like pancreatitis have also been implicated. Traumatic pseudoaneurysms are generally associated with high-velocity blunt trauma; however, penetrating trauma, as in our patient, can also lead to delayed vascular injury (3). Although the initial surgical repair of the liver injury may control active bleeding, small arterial injuries can be missed, evolving into pseudoaneurysms over time (9). In our case, the pseudoaneurysm likely developed from a partially injured segmental branch of the left hepatic artery that was not evident during the initial operation.

The true incidence of hepatic artery pseudoaneurysms remains low, with literature suggesting a prevalence of approximately 0.001% in the general population (10). However, their incidence is likely underestimated due to asymptomatic presentation or misdiagnosis. Most reported cases are linked to iatrogenic causes, with traumatic pseudoaneurysms comprising a smaller proportion. Among trauma cases, those due to blunt injuries are more frequently reported than those from penetrating injuries, possibly due to the operative nature of penetrating trauma that facilitates early identification and repair of vascular lesions (11). Our case is unique in that the pseudoaneurysm and associated APF developed despite surgical intervention, highlighting the need for continued postoperative surveillance even after seemingly successful repair.

The limited number of directly comparable reports makes it difficult to establish a standardized management algorithm for hepatic artery pseudoaneurysm associated with arterioportal fistula after penetrating trauma. Therefore, the present case should be interpreted within the broader context of post-traumatic hepatic artery pseudoaneurysms. Recent evidence suggests that hepatic pseudoaneurysm is uncommon after liver trauma but becomes more frequent with increasing injury grade. A systematic review and meta-analysis reported a pooled risk of 1.8%, with higher risk among grade III–V injuries and an average detection time of approximately 6 days after trauma. Similarly, a large trauma-center series found that hepatic pseudoaneurysms were often detected several days after injury, either through surveillance CT angiography or after symptoms such as bleeding, pain, or hemodynamic deterioration (12). These findings support the importance of maintaining suspicion for delayed vascular complications even after initial operative control of hepatic bleeding.

In the present case, the patient had a surgically repaired grade III left hepatic lobe laceration, followed by persistent blood-tinged drain output and epigastric pain despite hemodynamic stability. These subtle postoperative findings prompted additional imaging and ultimately led to the diagnosis of left hepatic artery pseudoaneurysm with arterioportal fistula. This clinical course is important because penetrating injuries are often explored surgically, and vascular lesions may be assumed to have been controlled intraoperatively. However, small segmental arterial injuries may evolve into delayed pseudoaneurysm or fistulous communication, particularly when the initial arterial wall injury is partial or temporarily tamponaded.

To better contextualize the rarity and management challenges of this condition, we summarized the available published reports and relevant post-traumatic hepatic pseudoaneurysm literature in Table 2. Because directly comparable cases of hepatic artery pseudoaneurysm with arterioportal fistula after penetrating trauma are limited, the table includes both closely related traumatic cases and broader post-traumatic hepatic pseudoaneurysm reports, focusing on mechanism of injury, clinical presentation, treatment strategy, need for repeat intervention, and postoperative outcome (Table 2).

**Table 2:** Focused comparison of published reports and contextual literature on traumatic hepatic artery pseudoaneurysm with or without arterioportal fistula.

Study/source	Mechanism or population	Vascular lesion	Presentation/diagnosis	Management strategy	Outcome/educational point
	Case series of 5 patients with acute hepatic arterioportal fistula after liver trauma; 127 high-grade penetrating liver injuries were reviewed.	Hepatic arterioportal fistula after high-grade liver trauma.	Most patients presented with hemorrhagic shock; diagnosis was made by postoperative or direct angiography/CT-based evaluation.	Operative hemorrhage control was often required first; definitive fistula treatment used endovascular techniques including coil embolization, Gelfoam, and Gelfoam combined with n-butyl cyanoacrylate.	All 5 patients had resolution on follow-up imaging. Supports multidisciplinary decision-making and tailored embolization based on hemodynamics and fistula flow.
	47-year-old man with penetrating knife injury to the liver.	Delayed hepatic pseudoaneurysm in a left hepatic artery branch; no arterioportal fistula reported.	CT on postoperative day 12 showed no pseudoaneurysm; follow-up CT on postoperative day 25 detected an asymptomatic pseudoaneurysm.	Coil embolization was performed.	Discharged without complications; no recurrence at 1 year. Demonstrates that penetrating-trauma pseudoaneurysm may appear late even after earlier negative CT.
Ahmed et al. (2021) (6)	23-year-old man with right thoracoabdominal gunshot wound and grade IV liver injury.	Ruptured right hepatic artery pseudoaneurysm with active extravasation.	Presented 2 weeks after injury with abdominal pain/lightheadedness, severe anemia, hemoperitoneum, and CTA evidence of active bleeding.	Damage-control laparotomy with packing followed by right hepatic artery coil embolization; planned re-exploration and closure followed.	Discharged stable with uneventful follow-up. Highlights need for hybrid operative/endovascular management when rupture or shock is present.
	10-year-old boy after high-speed motor vehicle collision.	Traumatic hepatic artery pseudoaneurysm with arterioportal fistula.	Complex vascular injury identified after blunt trauma.	Combination of endovascular stent graft and coil embolization using flow control with balloon remodeling.	Shows that combined endovascular techniques may be useful for complex/high-flow lesions when parent-artery preservation or flow control is important.
	21-year-old man after blunt abdominal trauma.	Two hepatic pseudoaneurysms followed by a separate arterioportal shunt on interval imaging.	Follow-up MDCT on day 10 showed pseudoaneurysms; follow-up CT on day 17 identified an arterioportal shunt and perfusion abnormality.	Superselective coil embolization of pseudoaneurysms, followed by angiographic confirmation and coil embolization of the arterioportal shunt.	Illustrates value of serial imaging, because pseudoaneurysm and arterioportal shunt may be detected at different time points.
	Retrospective Level 1 trauma-center cohort of 634 hepatic injuries.	Post-traumatic hepatic pseudoaneurysm.	18 pseudoaneurysms were documented; median identification was 6.5 days. Incidence increased with injury grade: grade III 1.8%, grade IV 6.5%, grade V 17.3%.	Surveillance CT angiography detected some asymptomatic lesions; symptomatic lesions prompted CT angiography and intervention.	Supports interval CT angiography 5–7 days after injury, especially for high-grade injuries.
Duncan et al. (2023) (12)	Systematic review and meta-analysis of hepatic pseudoaneurysm risk after liver trauma.	Post-traumatic hepatic pseudoaneurysm.	Pooled risk was 1.8%; average detection time was about 6 days. Risk increased with AAST grade, especially grades III-V.	Not an intervention study; evaluates surveillance need by injury severity.	Provides broader evidence base for surveillance imaging in grade III-V liver trauma.
Present case	25-year-old man with penetrating stab wound to the right upper quadrant/epigastrium and surgically repaired grade III left hepatic lobe laceration.	Left hepatic artery pseudoaneurysm with arterioportal fistula.	Persistent blood-tinged drain output and epigastric/right upper quadrant pain despite hemodynamic stability; CT suggested a vascular lesion and angiography confirmed pseudoaneurysm with fistulous communication.	Initial Gelfoam embolization failed; definitive coil embolization via right femoral access excluded the pseudoaneurysm and resolved the fistulous communication.	Adds a rare example of delayed HAP with APF after penetrating trauma despite initial operative repair; emphasizes early vascular imaging and definitive endovascular therapy when subtle postoperative signs persist.

AAST, American Association for the Surgery of Trauma; APF, arterioportal fistula; CT, computed tomography; CTA, computed tomography angiography; HAP, hepatic artery pseudoaneurysm; MDCT, multidetector computed tomography.

Overall, the available literature suggests that management should be individualized according to hemodynamic stability, lesion anatomy, fistula flow dynamics, and available endovascular expertise. In stable patients, transarterial embolization is generally favored because it provides both diagnostic confirmation and definitive treatment, whereas surgery remains important in unstable patients or when endovascular therapy is unavailable or unsuccessful.

The pathophysiology of pseudoaneurysm formation involves a focal disruption of the arterial wall, often sparing the adventitia or relying on surrounding tissues to contain the hemorrhage (13). This leads to the formation of a pulsatile hematoma in communication with the arterial lumen. Over time, increased arterial pressure can cause the pseudoaneurysm to expand or rupture. In certain cases, erosion into adjacent venous structures may occur, resulting in arteriovenous or arterioportal fistula formation (14). An arterioportal fistula creates a pathological shunt between the hepatic artery and portal vein, allowing high-pressure arterial blood to flow directly into the low-pressure portal system. This abnormal shunting can lead to complications such as portal hypertension, variceal bleeding, hepatomegaly, and ascites (15). In our patient, the pseudoaneurysm was located in a branch of the left hepatic artery and had eroded into both the portal and hepatic veins, creating a complex vascular communication that required endovascular intervention.

Clinically, hepatic artery pseudoaneurysms are highly variable in presentation. Many remain asymptomatic and are discovered incidentally. Symptomatic cases may present with right upper quadrant or epigastric pain, gastrointestinal bleeding (e.g., hematemesis or melena), jaundice, or hemodynamic instability if rupture occurs (8). In cases with arterioportal fistulae, signs of portal hypertension such as ascites, splenomegaly, or esophageal varices may be evident. Hemobilia, characterized by the classic triad of right upper quadrant pain, jaundice, and upper gastrointestinal bleeding, may occur when the biliary system is involved (16). In our case, the patient's symptoms were relatively nonspecific, including ongoing epigastric pain and serosanguinous drain output. These findings were initially subtle but ultimately led to further investigation that identified the underlying vascular abnormality.

Imaging plays a critical role in the diagnosis of hepatic pseudoaneurysms. Color Doppler ultrasonography may demonstrate swirling, bidirectional flow within the pseudoaneurysm sac, although sensitivity may be limited for small or deep lesions (8). Contrast-enhanced CT, particularly CT angiography, offers excellent spatial resolution and is often the first-line modality. However, digital subtraction angiography (DSA) remains the gold standard due to its diagnostic accuracy and the ability to proceed with endovascular treatment during the same session (17). In this case, contrast-enhanced CT initially raised suspicion for a vascular lesion, and DSA confirmed both the pseudoaneurysm and arterioportal fistula, allowing for targeted coil embolization.

The management of hepatic artery pseudoaneurysms depends on their size, location, clinical presentation, and presence of complications such as fistulae. Traditionally, surgery was the mainstay of treatment, particularly for unstable patients or ruptured pseudoaneurysms. However, advances in interventional radiology have made endovascular approaches the preferred option in most stable cases (18). Techniques include coil embolization, the use of Gelfoam or other embolic agents, and placement of covered stents (19). In our patient, initial embolization with Gelfoam was unsuccessful, likely due to the high-flow dynamics of the fistula. Coil embolization through the femoral artery provided definitive management, resulting in complete exclusion of the pseudoaneurysm and obliteration of the fistulous communication.

The management of hepatic artery pseudoaneurysms, particularly when associated with arterioportal fistula, should be individualized according to hemodynamic status, lesion size and location, fistula flow dynamics, arterial anatomy, clinical presentation, and the need to preserve hepatic arterial perfusion. Traditionally, surgery was the mainstay of treatment, especially in unstable patients or those with rupture and uncontrolled hemorrhage; however, advances in interventional radiology have made endovascular approaches the preferred option in most hemodynamically stable patients because angiography allows diagnostic confirmation and definitive treatment during the same session (18). Available endovascular techniques include coil embolization, temporary embolic agents such as Gelfoam, liquid embolic materials, thrombin injection in selected cases, and covered stent placement when preservation of the parent artery is required (19). After treatment, follow-up care is important to confirm technical success, detect recurrence, and monitor for ongoing bleeding, liver function abnormalities, or complications related to portal hypertension (20, 21). In addition, contemporary reports emphasize that treatment selection should consider the location of the pseudoaneurysm, fistula flow dynamics, arterial accessibility, and the feasibility of preserving hepatic arterial perfusion (22). Previous reports of traumatic hepatic artery pseudoaneurysm with arterioportal fistula have described successful treatment using transarterial embolization, repeat embolization, or combined stent-graft and coil techniques, depending on lesion anatomy and flow characteristics (23). In our patient, initial embolization with Gelfoam was unsuccessful, likely because the fistulous communication created a relatively high-flow vascular lesion. Definitive coil embolization through the femoral artery provided controlled and durable occlusion of the feeding arterial branch and pseudoaneurysm sac, resulting in complete exclusion of the pseudoaneurysm and obliteration of the fistulous communication.

In our patient, initial Gelfoam embolization failed, likely because the fistulous communication created a relatively high-flow vascular lesion. Definitive coil embolization was therefore selected to provide more controlled and durable occlusion of the feeding arterial branch and pseudoaneurysm sac. Post-embolization angiography confirmed exclusion of the pseudoaneurysm and resolution of the fistulous communication, and the patient improved clinically with resolution of vomiting, reduction of drain output, and improvement in abdominal pain.

This case is significant because it highlights a rare and potentially life-threatening vascular complication that developed despite appropriate initial surgical management of a penetrating liver injury. It underscores the importance of maintaining a high index of suspicion for delayed vascular injuries in trauma patients, especially those with unexplained postoperative symptoms. Moreover, it reinforces the value of advanced imaging and endovascular techniques in both diagnosing and treating such lesions. While hepatic artery pseudoaneurysms remain uncommon, prompt recognition and minimally invasive intervention can prevent severe complications and optimize patient outcomes. Our case contributes to the limited literature on post-penetrating hepatic pseudoaneurysms and supports the growing role of endovascular therapy in trauma-related vascular complications.

## Conclusion

Hepatic artery pseudoaneurysm with arterioportal fistula is a rare but potentially life-threatening complication of liver trauma, particularly following penetrating injury. Although often underdiagnosed due to nonspecific clinical features, early recognition through appropriate imaging and timely intervention is critical to prevent serious outcomes such as rupture or portal hypertension. This case highlights the importance of maintaining a high index of suspicion for delayed vascular complications even after initial surgical repair. Endovascular coil embolization remains a safe, effective, and minimally invasive treatment option that can lead to excellent clinical outcomes. Structured follow-up and multidisciplinary coordination are essential to ensure optimal patient recovery and prevent recurrence.

## Data Availability

The original contributions presented in the study are included in the article/Supplementary Material, further inquiries can be directed to the corresponding authors.
